# The microbial nexus: linking arsenic biogeochemistry with greenhouse gas emissions

**DOI:** 10.3389/fmicb.2026.1818899

**Published:** 2026-05-29

**Authors:** Zhengyu Wu, Mengqi Li, Chenhao Liu, Ran Luo, Tenglong Song, Pengna Zhang, Yanhong Wang, Ping Li

**Affiliations:** 1 State Key Laboratory of Geomicrobiology and Environmental Changes, China University of Geosciences, Wuhan, China; 2 School of Environmental Studies, China University of Geosciences, Wuhan, China; 3 Hubei Key Laboratory of Yangtze Catchment Environmental Aquatic Science, China University of Geosciences, Wuhan, China

**Keywords:** arsenic transformation, biogeochemical cycling, coupling mechanisms, greenhouse gases, microorganisms

## Abstract

Microorganisms play a pivotal role in driving both arsenic (As) biogeochemical cycling and the significantly anomalous greenhouse gas (GHG) levels frequently observed in naturally high-As environments; however, the mechanistic coupling between these two processes remains insufficiently characterized. This review presents a comprehensive synthesis of the interplay between microbial transformations of As (oxidation, reduction, and methylation/demethylation) and three major greenhouse gases—CH_4_, N_2_O, and CO_2_. We first summarize the key microbial taxa and molecular mechanisms governing As redox transformation, CH_4_ oxidation and methanogenesis, autotrophic CO_2_ fixation, and denitrification-driven N_2_O production. Building on this mechanistic foundation, we elucidate four experimentally and environmentally validated linkages, including (1) direct methane oxidation coupled with As(V) reduction (AOM-AsR), highlighting the molecular mechanisms that drive arsenic mobilization during anaerobic/aerobic methane oxidation and their environmental implications; (2) direct As(III) oxidation coupled with denitrification, linking the As and nitrogen cycles, wherein incomplete denitrification acts as a significant biological source of N_2_O; (3) direct As(III) oxidation driving autotrophic carbon fixation, offering a potential regional net carbon sink across diverse environments; and (4) indirect feedbacks mediated by As methylation/demethylation and geochemical mobilization, where detoxification-driven shifts in As speciation and local toxicity indirectly regulate downstream methanogenic communities and CH_4_ fluxes. Finally, we identify critical knowledge gaps regarding specific molecular pathways and multi-element interactions underlying these microbially driven couplings, and propose future research directions centered on deeper mechanistic elucidation. Overall, this review provides a robust scientific foundation for understanding the complex interplay between As biogeochemistry and GHG dynamics in specific environmental niches.

## 1 Introduction

Excessive emissions of greenhouse gases (GHG) and toxic element contamination constitute two of the most formidable environmental challenges of the modern era. Since the onset of the Industrial Revolution, atmospheric concentrations of carbon dioxide (CO_2_), methane (CH_4_), and nitrous oxide (N_2_O) have risen by over 47, 158, and 23%, respectively (IPCC, 2023). These trace gases are the primary drivers of the enhanced greenhouse effect, with CH_4_ and N_2_O exhibiting global warming potentials approximately 27.9 and 273 times greater than that of CO_2_, respectively (IPCC, 2023). Concurrently, the highly toxic metalloid arsenic (As) is ubiquitously distributed in global aquatic and terrestrial systems due to natural and anthropogenic inputs (e.g., mining, smelting, and pesticide application) and consequently threatens ecological integrity and public health (Niazi et al., 2017; Sher and Rehman, 2019). Although As transformation and GHG cycling have traditionally been treated as separate research domains, the recent frequent detection of anomalous GHG concentrations in various As-rich environments has highlighted their potential intersection.

Microbial metabolism acts as the vital bridge intrinsically coupling these two biogeochemical processes. In the As cycle, microbes dictate the metalloid’s speciation, mobility, and toxicity through oxidation, reduction, methylation, and demethylation (Yamaguchi et al., 2011; Zhang S. Y. et al., 2015). In parallel, they govern essential GHG dynamics via methanogenesis and CH_4_, N_2_O-producing denitrification, carbon fixation and organic matter decomposition (Hanson and Hanson, 1996; Knittel and Boetius, 2009; Liang et al., 2017; Ni et al., 2023). Over the past decades, substantial progress has been made in elucidating the microbial mechanisms underlying these biogeochemical cycles. In recent years, accumulating evidence reveals that microorganisms extensively bridge the As and GHG cycles via direct bioenergetic couplings as well as indirect ecological mechanisms. For instance, anaerobic As(III) oxidation coupled with nitrate (NO_3_^−^) reduction often triggers N_2_O emissions via incomplete denitrification (Zhang J. et al., 2015), whereas As(V) reduction linked to CH_4_ oxidation actively consumes CH_4_ (Shi et al., 2020; Shi et al., 2022); chemoautotrophic As(III) oxidation drives CO_2_ fixation, contributing to local primary production (Yamamura and Amachi, 2014). Collectively, microbial As transformations significantly impact regional GHG budgets, while the metabolic processes driving GHG cycling reciprocally regulate As speciation and mobility.

Despite these crucial findings, a systematic understanding of these coupling mechanisms remains fragmented across environmental microbiology, geochemistry, and climate science. To bridge this knowledge gap, this review aims to synthesize the key microbial taxa and molecular mechanisms involved in As redox transformations and GHG metabolism. Furthermore, it systematically elucidates the direct metabolic linkages and indirect ecological interactions between As biogeochemistry and C/N-driven GHG emissions, highlighting critical research gaps to guide future interdisciplinary research directions. This review provides a theoretical basis for the targeted management of As-contaminated environments, facilitating strategies that simultaneously mitigate As mobility and reduce local GHG emissions.

## 2 Mechanisms of As metabolism and greenhouse gas (CO_2_, CH_4_, N_2_O) metabolism in microorganisms

### 2.1 Microbial As oxidation, reduction and methylation

The toxicity and mobility of As are fundamentally governed by its chemical speciation. Generally, inorganic As species are significantly more toxic than their organic counterparts, with arsenite (As(III)) exhibiting far greater toxicity than arsenate (As(V)) and most methylated species (Gebel, 2001; Kamiya et al., 2013). Mechanistically, As(III) causes irreversible cellular damage by binding to critical thiol groups, whereas As(V) acts as a phosphate analog, competitively disrupting metabolic pathways (Smith et al., 1992; Cervantes et al., 1994). Beyond toxicity, speciation determines its environmental behavior. Uncharged As(III) typically exhibits weaker adsorption to soil minerals and higher mobility at circumneutral pH compared to As(V), which is readily immobilized by mineral surfaces like iron oxides (Bissen and Frimmel, 2003). Because abiotic transformations of As are kinetically restricted under typical environmental conditions, microorganisms serve as the key drivers governing As speciation and fate in nature (Lu et al., 2022).

**Figure 1:**
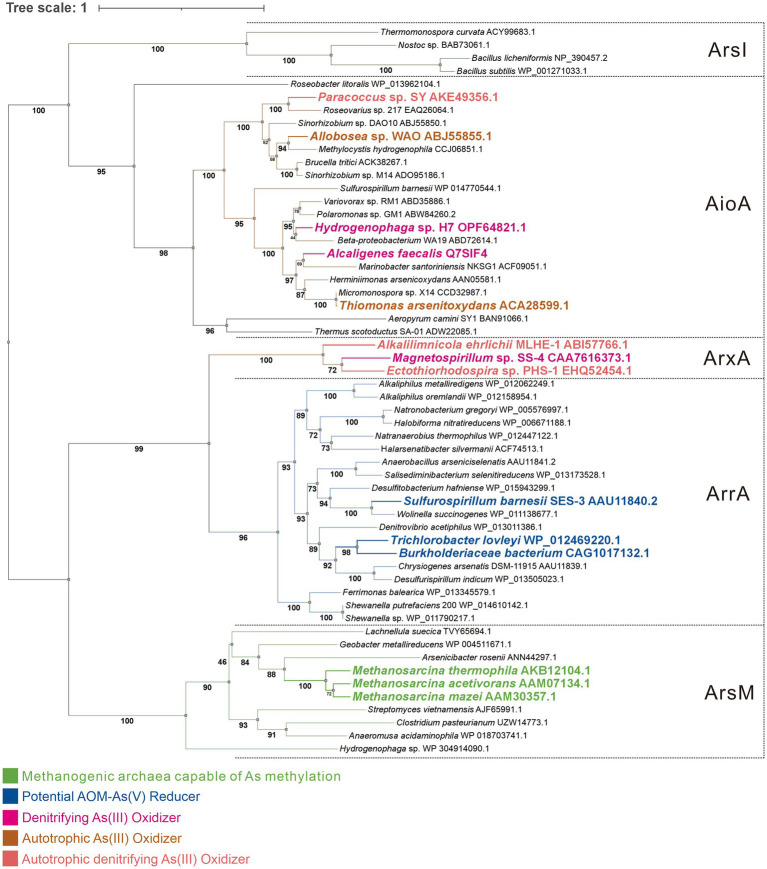
Maximum likelihood phylogenetic tree constructed from amino acid sequences of enzymes involved in arsenic transformation. Detailed methods are provided in the Supplementary material.

Microbial As(III) oxidation is mediated by two phylogenetically related oxidase systems: the facultative aerobic Aio system and the anaerobic Arx system. The Aio system, first purified from *Alcaligenes faecalis* (Anderson et al., 1992) (Figure 1), involves a periplasmic enzyme belonging to the dimethyl sulfoxide (DMSO) reductase family (Ellis et al., 2001). Its catalytic core comprises a large molybdopterin-dependent subunit (AioA) containing a [3Fe-4S] cluster and a small subunit (AioB) containing a [2Fe-2S] cluster. In some Aio-type arsenite-oxidizing bacteria, the catalytic core additionally includes a periplasmic cytochrome c552 (AioC/CytC), a TorD-like molybdenum enzyme chaperone (AioD), or a molybdenum biosynthesis enzyme (MoaA), exhibiting diverse compositions depending on the bacterial strain(Lieutaud et al., 2010; Freel et al., 2015; Wu Y. F. et al., 2023). Transcription of these genes is regulated by a sensory complex comprising the As(III)-binding periplasmic protein (AioX), the histidine kinase (AioS), and the transcriptional regulator (AioR), which initiate downstream gene transcription through a series of phosphorylation events (Sardiwal et al., 2010). Arx system, first characterized in *Alkalilimnicola ehrlichii* MLHE-1, is an obligately anaerobic arsenite oxidase system (Zargar et al., 2010). Compared to the Aio system, the Arx system is phylogenetically more closely related to the Arr arsenate reduction system. The composition of the Arx anaerobic arsenite oxidase shares certain similarities with the Aio system, comprising a molybdopterin-dependent oxidoreductase (ArxA), one or two small subunit containing a [4Fe-4S] cluster (ArxB), a membrane-anchoring protein (ArxC), and a TorD-like molybdenum enzyme chaperone (AioD), along with an upstream transcriptional regulatory gene cluster similar to that of the Aio system (Zargar et al., 2010). To date, only a restricted group of As-oxidizers are known to harbor the *arx* gene cluster (Figure 1), predominantly comprising *Gammaproteobacteria* isolated from extreme environments such as hot springs (Zargar et al., 2012).

Microbial As(V) reduction proceeds via two distinct mechanisms: dissimilatory reduction (respiratory reduction) and cytoplasmic detoxification (Figure 1). Dissimilatory As(V)-reducing bacteria utilize As(V) as a terminal electron acceptor for bioenergetic respiration (Kumarathilaka et al., 2018; Sarwar et al., 2021). This process requires the respiratory arsenate reductase (Arr), a molybdenum-dependent enzyme homologous to Arx and Aio. The catalytic core consists of the molybdopterin-containing ArrA subunit with an iron–sulfur cluster and the [4Fe-4S] cluster containing ArrB subunit. In contrast, the detoxification pathway is governed by the *ars* operon. Upon entry into the cytoplasm via phosphate transporters, As(V) is reduced to As(III) by cytoplasmic reductase ArsC and subsequently extruded by the ArsB efflux pump (Chang et al., 2008). Through these metabolic pathways, As(V)-reducing microorganisms play a significant role in mobilizing As.

As methylation is a crucial microbial detoxification strategy that converts inorganic As into generally less cytotoxic pentavalent methylated species, such as monomethylarsonate (MMA) and dimethylarsinate (DMA). Following the intracellular reduction of As(V) to As(III), this biotransformation is predominantly catalyzed by the S-adenosylmethionine-dependent methyltransferase ArsM (Figure 1) (Aposhian et al., 2004; Kruger et al., 2013; Ben Fekih et al., 2018). Alternatively, methanogenic archaea can incidentally methylate As via a side reaction of the methyltransferase MtaA. Unlike ArsM, MtaA-mediated methylation utilizes methylcobalamin as a methyl donor and is not regulated by intracellular As(III) concentrations, thereby generating a broader diversity of methylated arsenicals, including volatile trimethylarsine (TMA) (Thomas et al., 2011). In sulfide-rich environments, these initial methylation steps by ArsM or MtaA serve as essential precursors for arsenic thiolation, leading to the formation of structurally complex methylated thioarsenates (e.g., MMMTA and DMMTA) (Wang et al., 2014; Chen and Rosen, 2016). Conversely, this process is biologically counterbalanced by As demethylation, catalyzed by the C-As lyase ArsI (Figure 1), which cleaves methyl groups from trivalent organoarsenicals (Qin et al., 2006; Ben Fekih et al., 2018).

Beyond the direct microbial metabolism, the cycling of As is closely related to other redox-active elements, primarily Fe, S, and Mn. In many environments, As is tightly adsorbed onto Fe(III) and Mn(IV) (oxyhydr)oxides. Consequently, microbial reduction of Fe and Mn dissolves these host minerals, indirectly mobilizing As into the aqueous phase (Glodowska et al., 2020). Besides, microbial sulfate reduction generates sulfide, which can either sequester As into solid-phase precipitates (e.g., orpiment) or form soluble thioarsenate species (Kerl et al., 2018; Planer-Friedrich et al., 2018). Energetically, the availability of these oxidized elements (Fe^3+^, Mn^4+^, SO_4_^2−^) provides thermodynamically favorable alternative electron acceptors. Microbes utilizing these acceptors outcompete methanogens and certain denitrifiers for organic substrates and H_2_, thereby indirectly reshaping the overall GHG emission profile of the ecosystem.

### 2.2 Autotrophic CO_2_ fixation and microbially mediated CO_2_ release

Autotrophic carbon fixation represents the fundamental microbial process of assimilating inorganic CO_2_ into cellular organic constituents using light or chemical energy. Because this assimilation is highly energy-demanding, the availability of inorganic electron donors such as reduced metals and metalloids (including As) acts as a critical metabolic regulator in non-photosynthetic environments (Boyle and Morgan, 2011). While microorganisms have evolved diverse enzymatic routes for CO_2_ assimilation (e.g., the Calvin-Benson-Bassham (CBB) and reductive tricarboxylic acid cycles (rTCA)), all these pathways share a fundamental requirement for substantial energetic input. Once fixed, this microbially assimilated carbon, often in the form of biomass and extracellular polymeric substances, fuels downstream heterotrophic food webs (Taubert et al., 2022).

Conversely, microorganisms also serve as the planet’s primary decomposers, driving the return of CO_2_ to the atmosphere via mineralization (Philippot et al., 2024). During aerobic respiration, organic substrates undergo initial breakdown via glycolysis before being completely oxidized to CO_2_ through the TCA cycle. In oxygen-limited environments, anaerobic respiration utilizes alternative electron acceptors such as NO_3_^−^, SO_4_^2−^, Fe/Mn oxides and As(V), while fermentation processes also contribute to CO_2_ release (Schimel and Schaeffer, 2012). Ultimately, the dynamic balance between autotrophic CO_2_ sequestration and heterotrophic mineralization dictates the net environmental CO_2_ flux (Liang et al., 2017).

### 2.3 Microbial CH_4_ oxidation and methanogenesis

Globally, over half of the produced CH_4_ is oxidized by microorganisms before reaching the atmosphere (Bastviken et al., 2002). This biological CH_4_ filter is driven by aerobic methane-oxidizing bacteria (MOB) in oxic zones and anaerobic methane-oxidizing archaea (ANME) in oxygen-depleted environments (Xu et al., 2021). While aerobic MOB utilize methane monooxygenases to assimilate CH_4_, as comprehensively detailed in dedicated reviews (Ross et al., 2019), ANME are of particular biogeochemical significance for metal and metalloid cycling. ANME mediate the anaerobic oxidation of methane (AOM) via a “reverse methanogenesis” pathway, relying on variants of the methyl-coenzyme M reductase (Mcr) enzyme (Raghoebarsing et al., 2006). Because AOM is thermodynamically constrained, ANME typically establishes syntrophic partnerships with specific terminal electron acceptor-reducing bacteria (Knittel and Boetius, 2009). Through direct interspecies electron transfer (DIET) or the exchange of intermediate metabolites, ANME efficiently transfer the electrons derived from CH_4_ oxidation to these syntrophic partners, which sequentially reduce sulfate, NO_3_^−^, or oxidized metals/metalloids (Knittel and Boetius, 2009; Raghoebarsing et al., 2006; Beal et al., 2009). Crucially, the unique capacity of these syntrophic consortia to channel electrons toward As(V) reduction serves as the fundamental prerequisite for the AOM-AsR coupling modes explored in the next section.

Conversely, biogenic CH_4_ production is exclusively performed by methanogenic archaea under strictly anoxic conditions. Methanogenesis primarily proceeds via acetoclastic, hydrogenotrophic, or methylotrophic pathways, utilizing acetate, H_2_/CO_2_, or methylated compounds, respectively (Fricke et al., 2006; Liu and Whitman, 2008; Thauer et al., 2008). Despite these diverse upstream pathways, all methanogens share the *Mcr* enzyme complex to catalyze the terminal step of CH_4_ formation. Consequently, the *mcrA* gene serves as a universal genetic biomarker for tracking both methanogenesis and AOM in environmental studies. Ultimately, the intricate balance between archaeal methanogenesis and syntrophy-driven AOM strictly dictates the net CH_4_ emissions from anaerobic ecosystems.

### 2.4 Microbial N_2_O production and reduction

Denitrification is widely recognized as the dominant microbial process driving global N_2_O emissions (Pajares and Bohannan, 2016). This respiratory pathway sequentially reduces NO_3_^−^ to dinitrogen gas (N_2_) via nitrite (NO_2_^−^), nitric oxide (NO), and nitrous oxide (N_2_O), catalyzed by the enzymes Nar/Nap, Nir, Nor, and NosZ, respectively. While N_2_O is an intermediate, incomplete denitrification frequently results in its release (Prosser et al., 2020). Genomic surveys reveal that the abundance of *nir* genes (producing N_2_O precursors) often exceeds that of *nosZ* by an order of magnitude, indicating a high genetic potential for net N_2_O emission in many ecosystems (Shan et al., 2021). Typical partial denitrifies lacking *nosZ* include genera such as *Nocardioides*, *Rhodococcus*, and *Actinomadura* (Muller et al., 2006; Pang and Wang, 2020).

Under anaerobic conditions, dissimilatory nitrate reduction to ammonium (DNRA) represents a significant alternative pathway for NO_3_^−^ reduction. Catalyzed by enzymes such as *NrfA*, DNRA reduces NO_3_^−^ to NO_2_^−^ and subsequently to ammonium (NH_4_^+^), thereby conserving nitrogen within the ecosystem rather than removing it as gas (Xu et al., 2024). While DNRA itself does not typically produce N_2_O, it competes with denitrifiers for NO_3_^−^, potentially influencing the overall N_2_O budget by modulating substrate availability for denitrification.

N_2_O is also generated as a byproduct of nitrification via the hydroxylamine oxidation pathway. During the oxidation of NH_4_^+^ to NO_3_^−^, the intermediate hydroxylamine (NH_2_OH) can be incompletely oxidized. Historically, it was proposed that hydroxylamine oxidoreductase (Hao) directly converts NH_2_OH to N_2_O (Arp and Stein, 2003). However, recent enzymatic evidence suggests that Hao produces NO, which is subsequently reduced to N_2_O via Nor or chemical decomposition of unstable intermediates (Kozlowski et al., 2014).

## 3 Microbial As cycling influences greenhouse gas emissions

**Figure 2:**
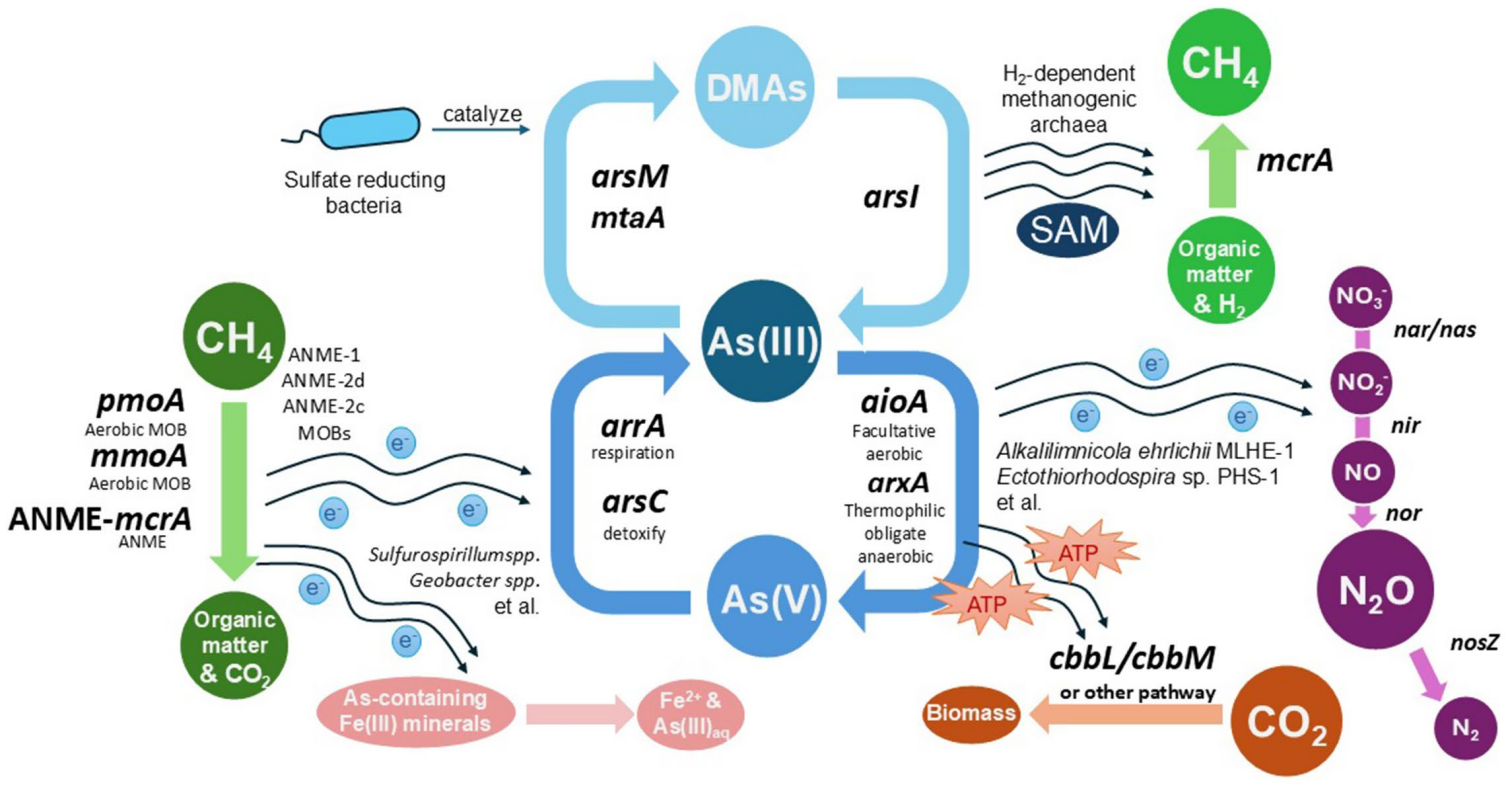
Coupling mechanism of arsenic redox transformation and (de)methylation with GHG emissions.

Microbial electron transfer intrinsically links the biogeochemical cycles of carbon, nitrogen, and arsenic. Building upon the previously discussed molecular mechanisms, this section categorizes the interactions between As cycling and GHG metabolism into two distinct dimensions. First, we examine direct metabolic couplings, where As serves as a direct electron donor or acceptor. This include CH_4_ oxidation coupled to As(V) reduction, N₂O-yielding As(III) oxidation-driven denitrification, and As(III)-driven autotrophic CO₂ fixation. Second, we explore indirect geochemical and ecological interactions that may affect As and GHG metabolism. This dimension addresses how the interplay with other redox elements especially Fe governs As mobility and microbial energetic competition, alongside the ecological impacts of As toxicity and methylation/demethylation on community restructuring. Collectively, these diverse coupling mechanisms illustrate the multifaceted nature of microbial interactions linking As biogeochemistry with greenhouse gas dynamics (Figure 2).

### 3.1 Direct metabolic coupling mechanisms

#### 3.1.1 Coupling of As(V) reduction with CH_4_ oxidation

**Figure 3:**
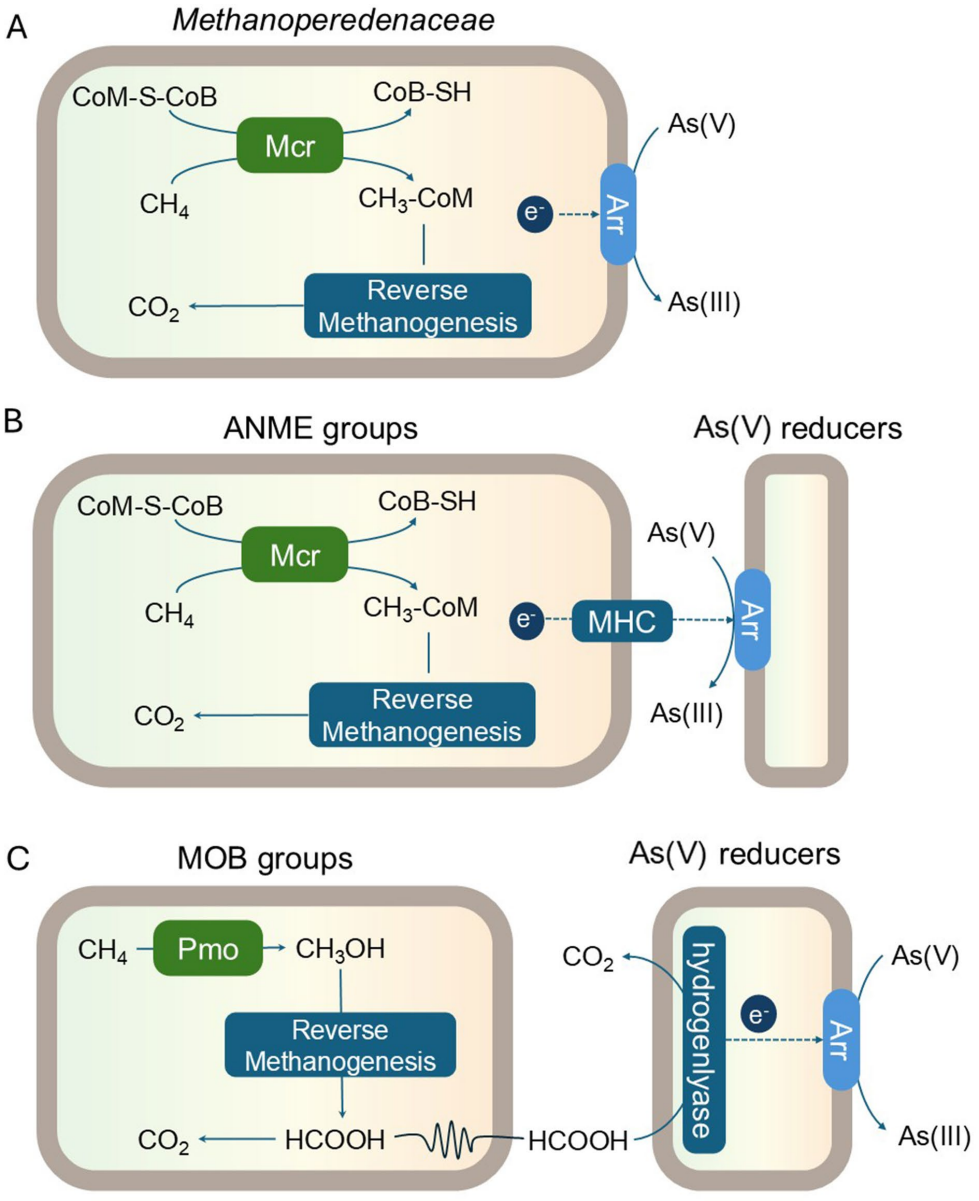
Conceptual mechanism of methane oxidation coupled with As(V) reduction across different microbial groups, adapted from Shi et al. (2020). **(A)** *Methanoperedenaceae* mediates respiratory As(V) reduction via intracellular electron transfer derived from CH_4_ oxidation; **(B)** Electron transfer between ANME groups and respiratory As(V) reducers drives CH_4_ oxidation and As(V) reduction; **(C)** Electron transfer between aerobic MOB and respiratory As(V) reducers drives CH_4_ oxidation and As(V) reduction.

Thermodynamically, CH_4_ oxidation coupled with As(V) reduction [AOM-AsR, CH_4_ + 4As(V) → CO_2_ + 4As(III), ΔG = −478 kJ/mol CH_4_] is highly favorable (Caldwell et al., 2008), representing a significant but often overlooked methane sink. Building upon molecular mechanisms, this coupling is increasingly recognized as a diverse process driven by distinct microbial consortia in different environmental niches (Figure 3).

In anaerobic environments such as wetlands, AOM-AsR is primarily driven by methanotrophic archaea (ANME) (Figure 3B). Studies have identified strong correlations between ANME lineages (ANME-1, ANME-2c, and ANME-2d) and Arr-type As(V)-reducing bacteria (e.g., *Sulfurospirillum*, *Geobacter*) (Figure 1), where ANME likely provides electrons to As(V) reducers via direct or indirect interspecies electron transfer (Shi et al., 2020, 2022). Moreover, archaea such as *Methanoperedenaceae*, which possess both CH_4_ oxidation pathways and the Arr enzyme, are capable of facilitating electron transfer *in vivo* for the coupling reaction (Figure 3A). Microcosm and ^13^C-labeling experiments have quantified the significance of this pathway, with ANME-mediated AOM-AsR contributing 26–49% of total As release in wetland soils, and as much as 33.87–80.76% in specific field and laboratory settings, with ANME-2d identified as the primary driver (Shi et al., 2020; Zhou et al., 2023). In contrast to wetlands, aerobic methane-oxidizing bacteria (MOB, e.g., *Methylocystis*, *Methylosinus*, *Methylomonas*) dominate CH_4_ oxidation in paddy soils. These MOB exhibit significant correlations with *Arr*-type As(V)-reducers, suggesting a potential metabolic syntrophy where methane oxidation facilitates an environment conducive to As reduction (Figure 3C, Shi et al., 2020, 2022).

The efficiency of these couplings is sensitive to environmental and biological stressors. For instance, elevated temperatures (33 °C) can inhibit AOM-AsR in paddy soils, potentially creating a positive climate feedback loop where warming-induced suppression of this methane sink exacerbates CH_4_ emissions (Zhou et al., 2025). Furthermore, biological pressures such as viral predation, including both extracellular free viruses and mitomycin C-induced prophages, can suppress As(III) mobilization by significantly reducing the abundance of key drivers like ANME-2d (Wang et al., 2025). From a management perspective, soil amendments such as iron-based passivators or low-molecular-weight organic acids have been shown to effectively inhibit AOM-AsR by modulating the functional activity of ANME communities, offering potential strategies for simultaneous CH₄ mitigation and As immobilization (Yang et al., 2022; Zhang et al., 2024).

#### 3.1.2 As(III) oxidation coupled denitrification

**Figure 4:**
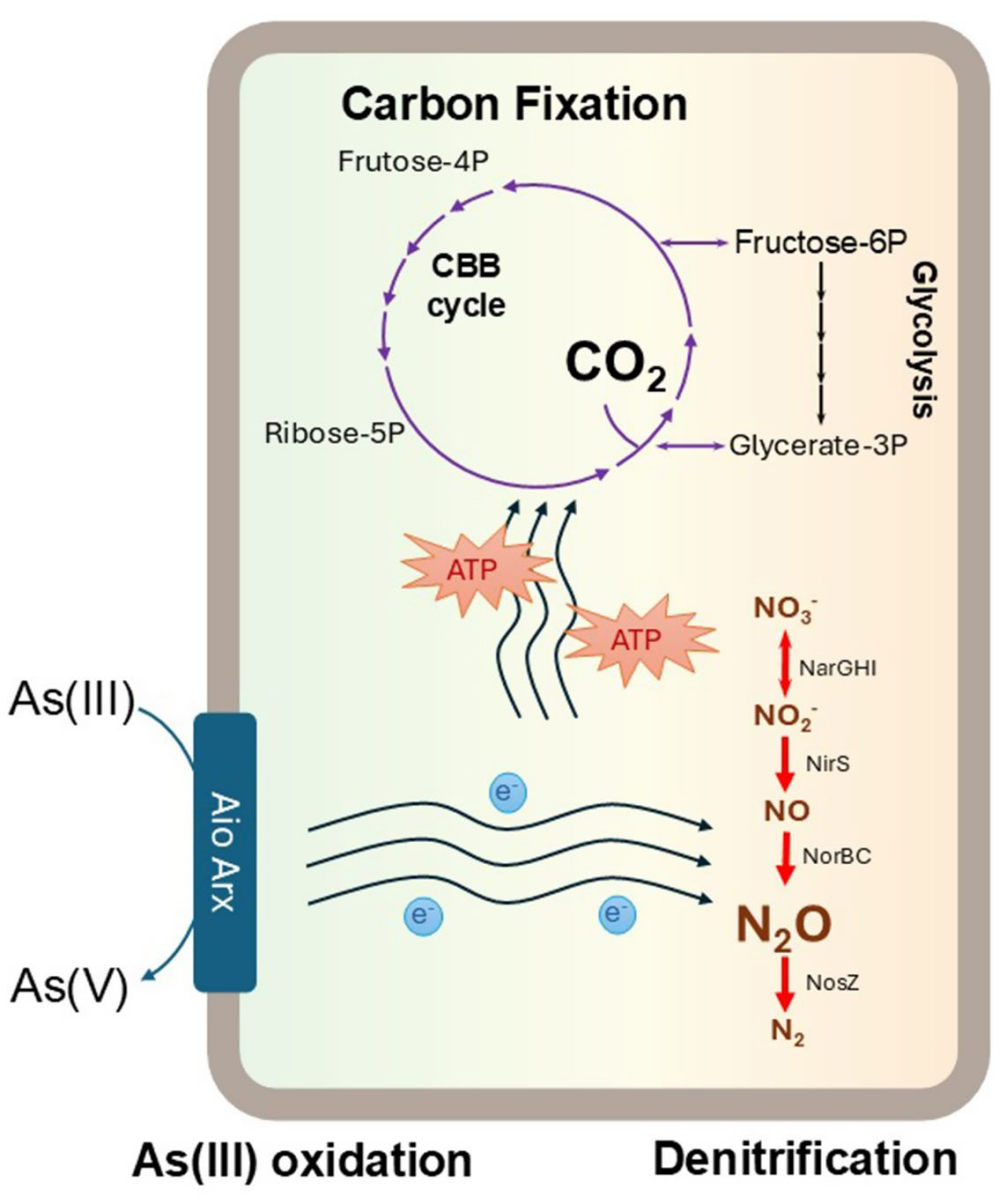
Coupling mechanism of arsenic oxidation, denitrification and carbon sequestration.

In anoxic environments where the highly mobile As(III) accumulates, NO_3_^−^ serves as a critical alternative electron acceptor for microbial respiration[5As(III) + 2NO_3_^−^ → 5As(V) + N_2_, ΔG = −117.3 kJ/mol As(III); 4As(III) + 2NO_3_^−^ → 4As(V) + N_2_O, ΔG ≈ −92 kJ/mol As(III)] (Sun et al., 2008). The coupling of anaerobic As(III) oxidation with denitrification provides vital bioenergetics for diverse microbes, but more importantly from a climate perspective, it acts as a direct driver of N₂O emissions (Figure 4).

During canonical denitrification, NO_3_^−^ is sequentially reduced to N_2_. However, As(III)-driven denitrification frequently results in incomplete denitrification and the subsequent accumulation of N_2_O. This accumulation may be primarily attributed to two factors. Firstly, many As(III)-oxidizing taxa possess truncated denitrification pathways lacking functional *nosZ* genes, with representative strains like *Alkalilimnicola ehrlichii* MLHE-1 even limited to partial reduction to NO_2_^−^ (Figure 1) (Oremland et al., 2002). Secondly, elevated As concentrations can directly inhibit the activity of nitrous oxide reductase (NosZ), creating a metabolic bottleneck that forces N_2_O pooling (Ding et al., 2025). This physiology is widespread and mediated by diverse taxa harboring either the *arx* or *aio* gene systems. Known drivers range from the anaerobic As(III)-oxidizer *Azoarcus* sp. DAO1 and facultative *Sinorhizobium* sp. DAO10 in As-contaminated soils (Rhine et al., 2006, 2007), to the Arx-type photoautotrophic *Ectothiorhodospira* sp. PHS-1 in hot springs (Kulp et al., 2008), and the Aio-type autotroph *Paracoccus* sp. SY in paddy soils (Zhang S. Y. et al., 2015) (Figure 1). Notably, even under aerobic conditions, certain strains like *Hydrogenophaga* sp. H7 can perform simultaneous As(III) oxidation and denitrification, actively converting soil nitrogen pools into gaseous forms (Fan et al., 2022) (Figure 1).

Beyond pure cultures, environmental metagenomics and microcosm studies have corroborates that this metabolic coupling actively reshapes N cycling *in situ*. In anaerobic paddy soil microcosms, As(III) oxidation rates strongly correlate with NO_3_^−^ concentrations, driven by key functional taxa of *Pseudogulbenkiania* (NO_3_^−^-reducers) and *Azoarcus* spp. (As-oxidizers), as evidenced by significant correlations between *aioA* and *narG* genes (Li et al., 2020; Li et al., 2023). Moreover, treatments such as fulvic acid-mediated photocatalysis (Zeng et al., 2024) and NO_3_^−^ addition in estuarine sediments (Ding et al., 2025) strongly stimulate this coupled process. In coastal sediments, opposing depth profiles of As and NO_3_^−^ in pore water further suggest active in situ coupling (Zou et al., 2025). This highlights a critical environmental risk where nitrogen fertilization in As-rich environments may inadvertently spike N_2_O emissions.

Emerging research is expanding the known boundaries of this coupling, revealing that interplay between As and N determines not just the production, but the net flux of N_2_O. For instance, newly identified pathways such as anaerobic ammonium oxidation (anammox) coupled with As reduction can promote simultaneous As release and further N₂O production (Liu et al., 2026). Metagenomic evidence from *Serratia* spp. hints at a novel link between As(III) oxidation and nitrogen fixation, a process that potentially involves N_2_O consumption (Li et al., 2022). Collectively, these discoveries underscore that the coupled biogeochemical cycling of As and N serves as a complex biological switch regulating both arsenic mobility and global N_2_O dynamics.

#### 3.1.3 As(III) oxidation coupled with CO_2_ fixation

For As(III)-oxidizers, the distinction between autotrophic and heterotrophic metabolism extends beyond carbon source utilization, as it fundamentally dictates their oxidation strategies and ecological roles. Heterotrophic As(III)-oxidizers oxidize As(III) primarily as a detoxification mechanism, requiring organic compounds for energy and biomass. In contrast, autotrophic or facultative autotrophic As(III) oxidizers harness the energy generated from electron transfer during As(III) oxidation to drive CO_2_ fixation (Figure 4). This metabolic divergence is sharply reflected in their responses to organic matter. Obligate heterotrophs, such as *Herminiimonas arsenicoxydans*, exhibit enhanced As(III) oxidation efficiency when supplied with organic carbon (e.g., glucose) or within biofilms containing complex organic matter (Muller et al., 2006). Conversely, for facultative autotrophs like *Anoxybacillus* sp. TCC9-4, while organic matter promotes growth, it significantly inhibits As(III) oxidation rates (Jiang et al., 2015).

Many of the anaerobic As(III)-oxidizers discussed previously, which couple oxidation with denitrification, are obligate or facultative autotrophs adapted to oligotrophic, low-redox environments. Prominent examples include the Arx-type *Alkalilimnicola ehrlichii* MLHE-1, the photoautotroph *Ectothiorhodospira* sp. PHS-1 (Oremland et al., 2002; Kulp et al., 2008), and the paddy soil isolate *Paracoccus* sp. SY (Oremland et al., 2002; Kulp et al., 2008; Zhang J. et al., 2015). In these organisms, the energy or reducing equivalents (electrons) derived from As(III) oxidation (often coupled with nitrate reduction) are channeled into CO_2_ fixation pathways such as the CBB cycle (Figure 4). This has been evidenced in acidic uranium mining environments, where coupling of anaerobic microbial As(III) oxidation with NO_3_^−^ reduction facilitates CO_2_ fixation and enhances local dissolved organic matter (DOM) content (Yu et al., 2025). Aerobic counterparts, such as *Thiomonas arsenitoxydans* strain 3AsT and *AlloBosea* sp. WAO (Figure 1), also employ this autotrophic strategy (Rhine et al., 2007; Slyemi et al., 2011).

However, while this As(III)-driven CO_2_ fixation is ecologically fascinating, its role in climate regulation must be interpreted cautiously and distinguished from the impacts of CH_4_ and N_2_O. As(III)-driven CO_2_ fixation largely does not constitute true primary production. Instead of contributing to long-term atmospheric carbon sequestration, it primarily drives short-term carbon cycling. The labile organic carbon newly synthesized by these autotrophs is rapidly scavenged by co-existing heterotrophs such as the As(V)-respiring bacteria discussed earlier, which subsequently mineralize the organic matter and re-release CO_2_ (Taubert et al., 2022). In summary, this metabolic dichotomy dictates the role of As(III) oxidation in cellular energy metabolism, but its macro-ecological impact is largely confined to sustaining localized microbial food webs. Understanding these strategies remains paramount for accurately predicting the biogeochemical behavior of As, even if their implications for global atmospheric GHG budgets are negligible compared to other coupled pathways.

### 3.2 Indirect ecological and geochemical interactions

#### 3.2.1 As methylation/demethylation and downstream community interactions

**Figure 5:**
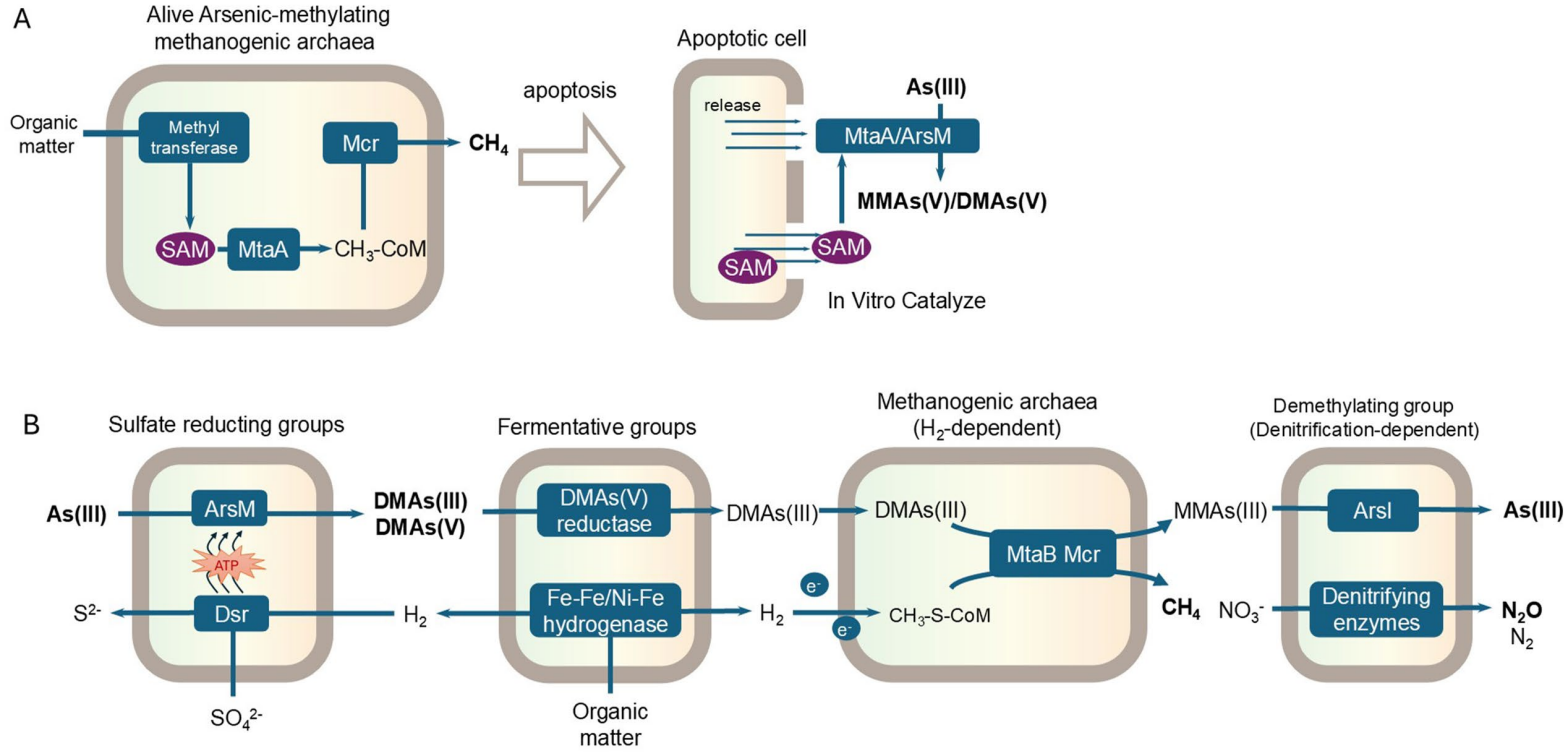
Coupling mechanism of As methylation/demethylation and methanogenesis. **(A)** As methylation catalyzed by CH_4_-producing metabolites from cellular residues of As-methylating methanogenic archaea; **(B)** Interaction mechanisms of co-mediated As methylation/demethylation coupled with methanogenesis by SRB group, hydrogen-producing group, methanogens, and denitrifying group.

Unlike As(V) respiration or As(III) oxidation, which directly couple As transformations to cellular bioenergetics, As methylation and demethylation are fundamentally detoxification mechanisms. Consequently, their influence on GHG fluxes (e.g., CH_4_) is largely indirect and highly context dependent. These transformations are mediated by a vast phylogenetic diversity of microorganisms. Beyond methanogens, a wide array of aerobic and anaerobic taxa, including sulfate-reducing bacteria (SRB) and other diverse microorganisms, play significant roles in the global cycling of methylated As species (Figure 5).

Within these functional microbial groups, the role of methanogens in As methylation has garnered specific attention due to their unique metabolic traits (Figure 5A). Arsenic methylation was first noted in *Methanosarcina mazei* (Figure 1), although this initial observation was attributed to reactions involving methanogenic cofactors rather than a dedicated As-methyltransferase (Thomas et al., 2011; Wuerfel et al., 2012). The definitive capacity for enzymatic As methylation was established in 2014, when the *arsM* gene was characterized in *Methanosarcina acetivorans* C2A (Wang et al., 2014) (Figure 1). Comparative studies by Viacava et al. (2020) indicated that while anaerobes generally exhibit weaker As methylation capacity than aerobes, *Methanosarcina mazei* Gö1 and *Methanosarcina acetivorans* C2A are notable exceptions. In recent years, Wang et al. (2023) isolated *Methanosarcina thermophila* TM-1 from hot spring sediment enrichments, demonstrating its capacity to produce highly toxic methylated thioarsenates (Figure 1). Furthermore, *Methanosarcina acetivorans* C2A was shown to sequentially methylate MMA to DMA and TMA, while simultaneously reducing As(V) under adequate electron donor supplementation (Liang et al., 2025).

In addition to methylation, demethylation further complicates the As cycle (Figure 5B). The combination of metagenomics and ^13^C-labeling experiments revealed that DMA demethylation involves multiple microbial groups, most notably SRB and methanogens (Chen et al., 2019). This was confirmed by the isolation of *Methanosarcina mazei* CZ1, the first reported demethylating methanogen (Chen et al., 2023).

Crucially, the connection between these bidirectional As transformations and CH_4_ production is indirect. Rather than acting as a direct metabolic coupling, methylation and demethylation alter local As speciation and toxicity. These chemical shifts, along with potential competition for methyl groups, regulate methanogen activity and shape the downstream microbial community structure. This intricate network of community interactions and indirect toxicity controls provides a more nuanced perspective for understanding CH_4_ emission dynamics in As-contaminated environments.

#### 3.2.2 Geochemical As mobilization and toxicity feedback in CH_4_ oxidation

**Figure 6:**
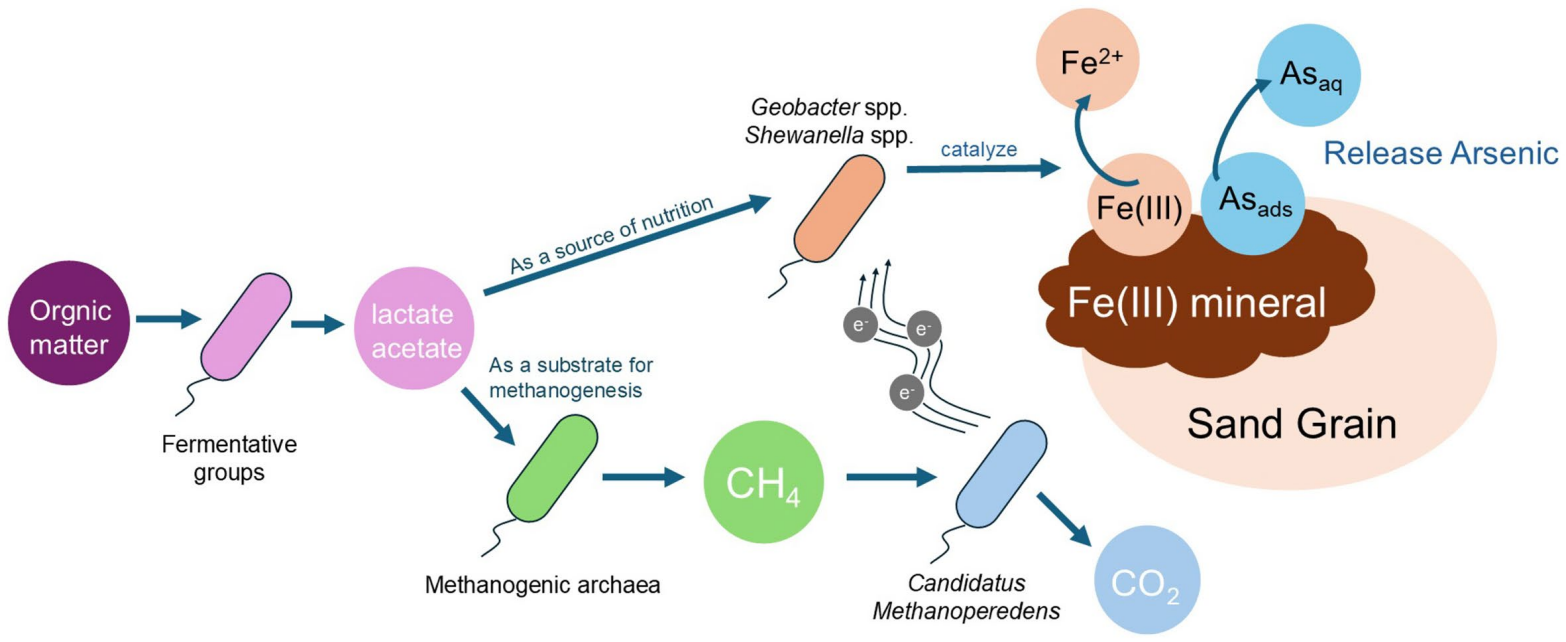
Coupling mechanism of As release caused by Fe(III) minerals reduction and CH_4_ oxidation, adapted from Glodowska et al. (2021), Viacava et al. (2020).

Beyond direct electron transfer, methane-oxidizing microbes exert profound indirect controls on As mobilization through complex geochemical interactions. Central to this mechanism is the CH_4_-driven reductive dissolution of As-bearing host minerals, particularly iron oxides. For instance, Glodowska et al. (2020) demonstrated that ANME-mediated Fe(III) reduction in aquifers acts as a critical biogeochemical trigger for As(III) release. Further high-resolution field evidence from the delta revealed that this process involves fermentation groups and methanogens providing nutrients and substrate for CH_4_-oxidizing bacteria and iron-reducing bacterial communities, which jointly mediate the reduction of arsenic-containing Fe(III) minerals, ultimately facilitating arsenic release (Figure 6, Glodowska et al., 2021). This indirect mobilization is metabolically versatile; subsequent research confirmed that enrichment of nitrate-dependent AOM (N-DAMO) also induces Fe(III) mineral reduction, thereby indirectly mobilizing As into groundwater (Glodowska et al., 2023). Beyond iron, this indirect mineral-driven linkage extends to sulfur cycling. In marine sediments, metagenomic analyses revealed strong correlations between genes for CH_4_ oxidation, As(V) reduction (*arrA*), and SO_4_^2−^ reduction (*dsrA*), linking active CH_4_ cycling to the dissolution of sulfide-bound As (Li et al., 2025).

Conversely, this geochemical mobilization initiates a critical toxicity feedback loop that indirectly constrains CH_4_ metabolism. As local As concentrations rise due to mineral dissolution, the ensuing toxicity can severely inhibit methanotrophic activity. Under high As stress (e.g., 30 mg/kg), a drastic 68.5% reduction in CH_4_ emissoins was observed, accompanied by a significant positive correlation between the As(V) detoxification gene (*arsC*) and the aerobic methanotrophy gene (*pmoA*) (Jiang et al., 2024). Collectively, these studies elucidate a complex, bidirectional indirect relationship: CH_4_ oxidation alters local geochemical gradients (via Fe and S reduction) to release As, while the resulting accumulation of As toxicity feeds back to restrict methanotroph activity and alter CH_4_ emission dynamics.

## 4 Outlook

While this review has synthesized the diverse direct metabolic linkages and indirect ecological interactions between microbially-mediated As cycling and GHG dynamics, it is crucial to explicitly acknowledge the limitations of the current synthesis. Current understanding is constrained by several factors, which subsequently define the critical frontiers for future research.

First, the existing literature remains largely phenomenological and qualitative. Future research must prioritize breakthroughs in elucidating quantitative mechanisms and determining precise energy/C/N fluxes. For instance, the contribution of autotrophic As(III) oxidation to carbon sinks in extreme environments (e.g., high-As groundwater and hot springs where As(III) oxidizers thrive) remains poorly constrained due to technical limitations. Future studies should integrate stable isotope probing (SIP) with metabolic flux analysis under near-natural conditions to delineate carbon partitioning through fixation pathways (e.g., the CBB cycle) and assess the true ecological role of As(III)-oxidizers as primary producers.

Second, current studies largely evaluate As-GHG linkages in isolation, limiting our understanding of complex natural systems. In natural soils and sediments, As transformations are inextricably linked to redox processes involving Fe and S (Glodowska et al., 2020; Wu Y. et al., 2023; Wu Z. et al., 2023). Consequently, multi-factorial experiments are urgently needed to clarify how these complex electron transfer networks compete, synergize, or inhibit one another, ultimately determining the magnitude of GHG fluxes. Furthermore, the quantitative dose–response relationships of indirect effects such as “toxicity stress” and long-term adaptive evolution mechanisms remain elusive. Specifically, how varying As concentrations modulate the expression and activity of key functional genes (e.g., *pmoA*, *mcrA*, *narG*, *cbbM*) warrants deeper investigation (Ahn et al., 2019). Addressing whether microbial communities face chronic inhibition or evolve novel metabolic strategies via horizontal gene transfer requires integrated research frameworks combining long-term microcosm experiments, in-situ monitoring, and time-series multi-omics.

Third, a significant limitation of the current synthesis is the spatial and environmental bias of the available data. Current knowledge is predominantly derived from specific, highly studied habitats such as paddy fields and hot springs. However, these interconnected biogeochemical processes likely occur extensively across diverse and understudied ecosystems, potentially governed by unique mechanisms. Future research must urgently expand its horizon to evaluate their global environmental significance. Priority should be given to frontier habitats, including high-As groundwater systems, acid mine drainage sites, coastal aquifers, and rapidly thawing Arctic permafrost. These systems represent hotspots of As mobilization, where distinctive physicochemical conditions (e.g., oligotrophy, low temperatures) may shape novel microbial functional guilds harboring as-yet-uncharacterized mechanisms. Simultaneously, advancing from local observations to global scales necessitates a proactive investigation of feedback effects within the context of global change. Factors such as warming, shifting precipitation, and permafrost thaw will profoundly perturb these established interactions by modulating microbial metabolic activities and community structures.

Ultimately, a profound understanding of these direct and indirect microbial mechanisms must serve the practical mission of addressing the dual environmental challenges of As pollution and GHG emissions. Translating scientific knowledge into environmental solutions requires technological innovation, particularly in engineering synthetic microbial communities and developing microbial electrochemical systems for targeted control. For instance, Chen et al. (2025) designed an integrated nitrate-birnessite-light system that leveraged mineral-microbe interactions to achieve complete immobilization of 0.1 mM As(III) while simultaneously reducing N_2_O, CH_4_, and CO_2_ emissions by 88, 77, and 19%, respectively. This study offers critical insights for developing strategies that couple As immobilization with GHG mitigation. In the broader context, embedding these “pollutant-GHG” synergistic effects into environmental assessment standards, agricultural management practices, and climate governance frameworks is essential. Such integration will be pivotal for translating microscopic microbial processes into tangible contributions toward the macroscopic sustainable development goals (Hallam et al., 2011).
