# Supplementary material for: The microbial nexus: linking arsenic biogeochemistry with greenhouse gas emissions
Source: Front Microbiol. 2026 May 29;17:1818899. doi: 10.3389/fmicb.2026.1818899 (PMC13262295; doi:10.3389/fmicb.2026.1818899)
Supplement: Supplementary file 3 [file Table_1.docx]

Supplementary Material

# Phylogenetic Tree Reconstruction Methods

Multiple sequence alignment of amino acid sequences was performed using the Muscle algorithm implemented in MEGA11, with parameter settings as specified in Graph 1. Detailed alignment results are provided in the supplementary materials (Alignment.xlsx and Alignment.txt).

| Option | Setting |
| --- | --- |
| Gap Open | -2.9 |
| Gap Extend | 0 |
| Hydrophobicity Multiplier | 1.2 |
| Max Memory in MB | 2048 |
| Max Iteration | 16 |
| Cluster Method (Iterations 1,2) | UPGMA |
| Cluster Method (Other Iterations) | UPGMA |
| Min Diag Length (Lanbda) | 24 |

Graph1 Settings of Muscle alignment

The acquired Alignment.mas file was analyzed using the Maximum Likelihood method in iqtree3.1.1 to construct a phylogenetic tree, with parameter settings as specified in Graph 2.

| Option | Setting |
| --- | --- |
| **CMD code** | **-st AA -m JTT -B 1000 --bnni -nt 4-st** |
| Statistical Method | Maximum Likehood |
| Test of Phylogeny | 1000 Times |
| Substitution Type | Amino Acid |
| Method | Jones-Taylor-Thornton model |
| ML Heuristic Method | Nearest-Neighbor-Interchange |
| Initial Tree for ML | Make initial tree automatically |
| Number of Threads | 4 |

Graph2 Settings of alignment analysis
